# A review of *Timandra* Duponchel, 1829 from China, with description of seven new species (Lepidoptera, Geometridae)

**DOI:** 10.3897/zookeys.829.29708

**Published:** 2019-03-11

**Authors:** Le Cui, Dayong Xue, Nan Jiang

**Affiliations:** 1 Key Laboratory of Zoological Systematics and Evolution, Institute of Zoology, Chinese Academy of Sciences, Beijing 100101, China Institute of Zoology, Chinese Academy of Sciences Beijing China; 2 University of Chinese Academy of Sciences, Shijingshan District, Beijing 100049, China University of Chinese Academy of Sciences Beijing China

**Keywords:** Morphology, Sterrhinae, taxonomy, Timandrini

## Abstract

The Chinese species of the genus *Timandra* Duponchel, 1829 are reviewed: 12 known species are reported. Seven new species are described from China, increasing the total number of *Timandra* species to 28: *T.distorta***sp. n.**, *T.adunca***sp. n.**, *T.quadrata***sp. n.**, *T.accumulata***sp. n.**, *T.viminea***sp. n.**, *T.robusta***sp. n.** and *T.stueningi***sp. n.** Diagnoses for all 19 Chinese species are provided, with illustrations of external features and genitalia.

## Introduction

The genus *Timandra* was originally established by Duponchel (1829) based on *Timandragriseata* Petersen, 1902, and he cited this species as *amataria* Linnaeus, 1761 due to a misidentification (Fletcher 1979). *Timandra* is the type genus of the tribe Timandrini. Its constituent species are distributed mainly in Asia, although the ranges of some extend to Europe and North America (Prout 1912–1916; Parsons et al. 1999; Hausmann 2004). The tribe Timandrini contains four genera: *Timandra*, *Synegiodes* Swinhoe, 1892, *Traminda* Saalmüller, 1891, and *Haematopis* Hübner, 1823. The general characters of Timandrini are as follows: the antennae of the male are bipectinate; the hind tibia of the male has no hair pencil; only one areole is present on the forewing, the veins R_2–4_ are always arising from the apex of the areole; the upper side of wings usually shows oblique transverse lines (except *Synegiodes*); in the male genitalia, the socii are often well developed, the valva is elongate and sometimes complex or bifurcate; in the female genitalia, the signum consists of a longitudinal ridge (Holloway 1997; Hausmann 2004; Sihvonen and Kaila 2004). However, there are no unique synapomorphies for Timandrini (Sihvonen and Kaila 2004), and the delimitation of tribe still needs further study.

Species belonging to *Timandra* are usually easy to be distinguished from other geometrids, although the Western Palaearctic *Scopulaimitaria* (Hübner, 1799) and some American species of *Arcobara* Walker, 1863 have similar pattern and wing shape. Prout (1913) briefly discussed the nomenclature of this genus and listed five species with concise descriptions. Later, he (Prout 1935) recorded ten *Timandra* species under the genus name *Calothysanis* Hübner, 1823 (an older name, which is currently treated as a subgenus of the genus *Scopula* Schrank, 1802; cf. Fletcher 1978, Hausmann 2004) and divided them into two sections according to the characters of the uncus. Kaila and Albrecht (1994) reviewed the European *Timandragriseata* group, and established three new combinations. Holloway (1997) recorded one species from Borneo, and summarized the characters of *Timandra* in detail. Sihvonen (2001) illustrated the everted vesicae of the male genitalia of *T.griseata* and *T.comae* and supported the conclusion of Kaila and Albrecht (1994) to treat them as valid species. Hausmann (2004) recorded three species from Europe with detailed descriptions and photos of the adults and the genitalia. Õunap et al. (2005) published a molecular phylogenetic study, which separates *T.griseata* and *T.comae* into different clades.

Twenty-one species have hitherto been recognized in *Timandra* (Parsons et al. 1999; Scoble and Hausmann 2007), including 12 species recorded in China (Prout 1912–1916, 1934–1939, 1920–1941; Chu and Xue 1988, 1992; Xue 1992a, 1992b; Wang 1997; Han and Xue 2002; Xue et al. 2002) . In the course of an inventory of the Sterrhinae of China (Cui et al. 2018a, 2018b; Xue et al. 2018), it became apparent that several new species of *Timandra* need to be described.

The purpose of this paper is to provide a survey of Chinese *Timandra* species, to describe the seven new species, and to provide diagnostic characters and illustrations of external features and genitalia of all Chinese species.

## Materials and methods

Specimens of *Timandra* are deposited in the following collections:

**BRCAS** School of Karst Science

**IZBE** Zoological-Botanical lnstitute of the Academy of Sciences, Tartu, Estonia

**IZCAS** Institute of Zoology, Chinese Academy of Sciences, Beijing, China

**NEFU** Northeast Forestry University, Harbin, China

**NHM** Natural History Museum, London, UK

**NHRS** Naturhistoriska Riksmuseet, Stockholm, Sweden

**NKU** Nankai University, Tianjing, China

**TFRI** Taiwan Forestry Research Institute, Taipei

**ZFMK** Zoologisches Forschungsmuseum Alexander Koenig, Bonn, Germany

Terminology for wing venation follows the Comstock-Needham system (Comstock 1918) as adopted for Geometridae by Scoble (1992) and Hausmann (2001); terminology for genitalia follows Pierce (1914, reprint 1976), Klots (1970), Nichols (1989), Kaila and Albrecht (1994), and Hausmann (2004). Photographs of the moths were taken with digital cameras. Composite images were generated using Auto-Montage software v. 5.03.0061 (Synoptics Ltd). The plates were compiled using Adobe Photoshop v. 7.0. Ink (Adobe Systems Software Ireland Ltd).

## Systematics

### Genus Timandra

Taxon classificationAnimaliaLepidopteraGeometridae

Duponchel, 1829

Timandra Duponchel, 1829: 105. Type species: Timandragriseata Petersen, 1902.
Bradyepetes Stephens, 1829: 44. Type species: Timandragriseata Petersen, 1902 [junior objective synonym of Timandra Duponchel].
Bradypetes Agassiz, 1847: 52 [emendation of Bradyepetes Stephens].

#### Generic characters.

***Head.*** Male antennae bipectinate, pectination covered with ciliae, usually black on basal part; female antennae filiform. Labial palpi with last segment narrow. Hind tibia in male not dilated, with two pairs of spurs, sometimes a black spot present at apex of one spur in each pair. *Venation*. Forewing with one areole; R_1_ arising distally or directly from apex of areole; R_5_ sometimes stalked with R_2–4_, sometimes not. Hindwing with Rs and M_1_ separate or shortly stalked, M_3_ and CuA_1_ separate. Forewing with acute apex, sometimes protruding outside, outer margin nearly straight or slightly arched; hindwing with outer margin forming a small protrusion on vein M_3_. Forewing with medial line oblique, arising from apex or subapex; postmedial line narrow, usually overlapping with medial line near apex. Hindwing with medial line straight. Postmedial line and discal spot often more distinct on underside than those on upperside.

***Male genitalia*.** Uncus often short digitiform, or slightly raised, sometimes dilated at tip. Socii usually well developed, sometimes absent. Valva with sclerotized strunctures and usually bifurcate; costa often sclerotized with a process; a short or tuberculate process usually present at base of valvula; a digitate arm usually extending from cleft between valvula and sacculus; sacculus often short, sometimes long and narrow, sometimes asymmetric between right and left valva. Juxta usually broad at base. Saccus often broad, sometimes concave. Aedeagus straight or curved; vesica with or without cornutus.

***Female genitalia*.** Seventh sternite usually strongly sclerotized and bifurcate on posterior margin, sometimes membranous. Papillae anales usually stout and short. Sterigma sometimes developed. Colliculum present. Ductus seminalis usually arising from posterior part of ductus bursae or apex of appendix bursae. Ductus bursae usually sclerotized on posterior part. Corpus bursae usually long and oval, membranous; signum with a longitudinal sclerite inside a slightly sclerotized plate, a pouch present on anterior part.

#### Diagnosis.

*Timandra* can be distinguished from other genera within Timandrini in the following external characters: the vein Sc+R_1_ of the hindwing is less strongly anastomosing with the cell in *Timandra* than in *Haematopis* (Prout 1931); the outer margin of the forewing below the apex often has an incision in *Traminda*, while it is straight in *Timandra*; the discal spot is reddish-brown or brown in *Timandra*, but consists of a black circle with a white center in *Synegiodes*. In the genitalia, *Timandra* can be easily distinguished by the combination of the following characters: the valva of the male genitalia is complex and often divided into two parts, with a slender digitiform process usually arises from the cleft between the valvula and the sacculus; the seventh sternite of the female is usually strongly sclerotized and bifurcated on the posterior margin.

#### Distribution.

Asia, Europe, and North America.

#### Host-plant.

Larvae have been recorded on Polygonaceae only (Holloway 1997; Hausmann 2004).

#### Remarks.

We found that the shape of the frons is variable in Chinese species of *Timandra*: slightly protruded in *T.oligoscia* Prout, 1918, *T.quadrata* sp. n., *T.robusta* sp. n., *T.dichela* (Prout, 1935), *T.griseata* Petersen, 1902, *T.extremaria* Walker, 1861, and *T.recompta* (Prout, 1930) (Fig. 38); fully protruded in *T.paralias* (Prout, 1935) and *T.distorta* sp. n. (Fig. 39); forming a rounded protrusion in *T.accumulata* sp. n. (Fig. 40), *T.apicirosea* (Prout, 1935), *T.ruptilinea* Warren, 1897, and *T.comptaria* Walker, 1863; with a sharp protrusion in *T.adunca* sp. n., *T.convectaria* Walker, 1861 (Fig. 41), and *T.correspondens* Hampson, 1895; protruded with an obtuse protrusion on the ventral margin in *T.viminea* sp. n. (Fig. 42); not protruding in *T.stueningi* sp. n.

The seventh sternite of the female is often not separated from the female genitalia, except in *T.griseata*, *T.convectaria* Walker, 1861, *T.viminea*, and *T.ruptilinea* Warren, 1897 in the present study (Figs 61, 65, 70, 72).

A male specimen from Hong Kong has a slightly different wing pattern from the other recognized species of *Timandra*, and may be a new species (Roger Kendrick pers. comm.). However, as only one specimen has been found, more specimens need to be collected and studied to allow for a full description.

### Timandra griseata

Taxon classificationAnimaliaLepidopteraGeometridae

Petersen, 1902

Figs 1 , 61

Timandra amata var. griseata Petersen, 1902: 239. Lectotype ♂, Estonia (IZBE).
Timandra serpentata ab. griseata : Prout 1913: 98.
Timandra griseata : Hausmann 2004: 390.

#### Diagnosis.

This species is very similar to *T.comae* Schmidt, 1931, but the combination of the following characters can distinguish them: the ground colour of the forewing is whitish in *T.griseata*, but often yellowish in *T.comae*; the surface of the wings is densely speckled with grey scales in *T.griseata*, but it is covered with brownish-grey speckles in *T.comae*; the average wingspan is larger and sexual size dimorphism is more accentuated in *T.griseata*. The appendix of the sacculus of the male genitalia is on average slightly broader at base in *T.griseata* than that in *T.comae*. In the female genitalia, the ductus bursae is stouter at the anterior half in *T.griseata*; the posterior appendix bursae of the ductus bursae arises at a bigger angle to the corpus bursae in *T.griseata* (Kaila and Albrecht 1994; Hausmann 2004; Õunap et al. 2005).

#### Material examined.

**CHINA: Xinjiang** (IZCAS): 1♀, Gongliu, Kuerdening, 1100 m, 26.VII.2017, coll. Cheng Rui.

#### Distribution.

China (Xinjiang), Europe.

#### Remarks.

This species is newly added to the fauna of China.

**Figures 1–19.**
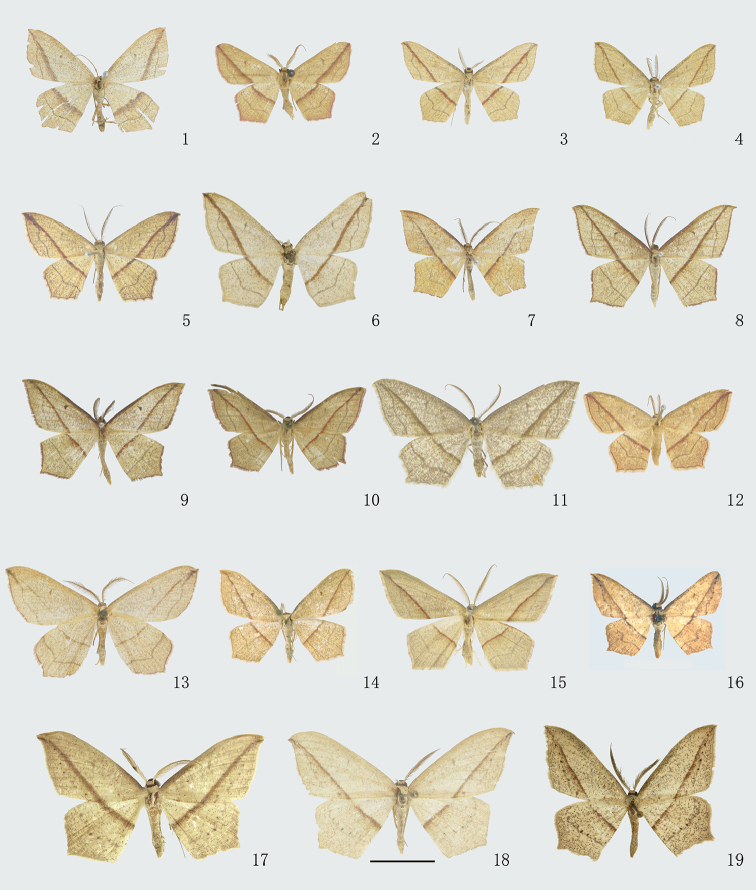
Adults of *Timandra*. **1***T.griseata*, female, Xinjiang **2***T.recompta*, male, Beijing **3***T.apicirosea*, male, Sichuan **4***T.distorta* sp. n., holotype, male, Tibet **5***T.dichela*, male, Hubei **6***T.synthaca*, holotype, male, Taiwan **7***T.convectaria*, male, Guangxi **8***T.correspondens*, male, Tibet **9***T.adunca* sp. n., holotype, male, Yunnan **10***T.quadrata* sp. n., holotype, male, Hubei **11***T.accumulata* sp. n., holotype, male, Yunnan **12***T.comptaria*, male, Zhejiang **13***T.paralias*, male, Hebei **14***T.viminea* sp. n., holotype, male, Yunnan **15***T.oligoscia*, male, Yunnan **16***T.ruptilinea*, male, Guangdong **17***T.extremaria*, male, Gansu **18***T.robusta* sp. n., holotype, male, Yunnan **19***T.stueningi* sp. n., holotype, male, Taiwan. Scale bar: 1 cm.

### Timandra recompta

Taxon classificationAnimaliaLepidopteraGeometridae

(Prout, 1930)

Calothysanis amata recompta Prout, 1930: 297. Holotype ♂, Russia: Ussuri [Ussuri region], Chabarovsk, Ussuri railway (NHM).
Timandra amataria myokosana Bryk, 1948: 159, pl. 7, fig. 9. Holotype ♂, Korea: Myokosan (NHRS).
Timandra recompta : Kaila and Albrecht 1994: 461.

#### Remarks.

At present, this species comprises three subspecies; other two subspecies are distributed in Japan and on Kurile Islands (Parsons et al. 1999).

### Timandra recompta recompta

Taxon classificationAnimaliaLepidopteraGeometridae

(Prout, 1930)

**Figures 20–27.**
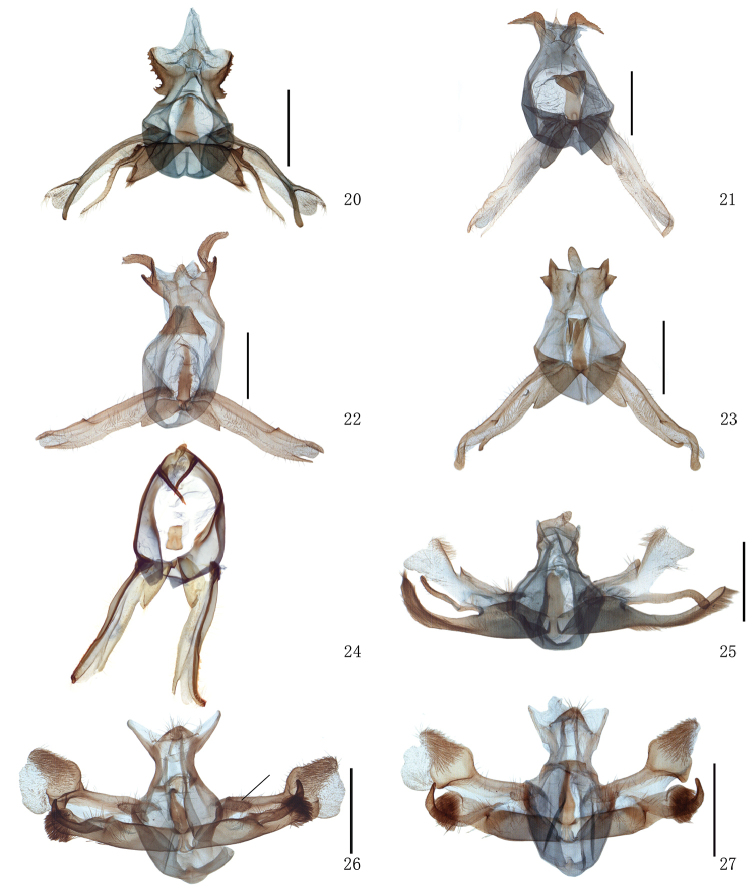
Male genitalia of *Timandra*. **20***T.recompta*, Heilongjiang **21***T.apicirosea*, Sichuan **22***T.distorta* sp. n., holotype, Tibet **23***T.dichela*, Hubei **24***T.synthaca*, male, Taiwan (without scale) **25***T.convectaria*, Sichuan **26***T.correspondens*, black bar shows the tuberculate process at the base of the valvula, Tibet **27***T.adunca* sp. n., holotype, Guangxi. Scale bar: 1 mm.

Figs 2 , 20 , 38 , 43 , 62

#### Diagnosis.

This species can be distinguished from its congeners by the following characters: the frons is slightly protruded; the pinkish-red shadow is present along the medial line and on the outer margin of both wings; in the male genitalia, the socii have a strongly serrate margin; the valvula has a narrow digitiform process subapically at the costa; the sacculus is short and with a pointed apex; the cornutus is short and stout with tiny spines. The female genitalia are similar to those of *T.griseata*, but the posterior margin of the seventh sternite is concave centrally.

#### Material examined.

**CHINA: Heilongjiang** (IZCAS): 3♀, Yichun, 15.IV.1957, 3.IX.1970; 16♂, Yichun, Dailing, 390 m, 24.VI.1957, 2.VIII–5.IX.1957, 24.VII.1958, 6.VII–2.VIII.1959, 8.VI.1962, coll. Bai Jiuwei et al.; 3♂1♀, Yichun, Wuyiling, 30–31.VIII.1970; 1♀, Yabuli, 29.VII.1939; 1♀, Mishan, Errenban, 11.VIII.1970. **Liaoning** (IZCAS): 1♂, Xinjin, 1954, coll. Ou Bingrong; 1♂, Anshan, Qian Shan, 30.VII–4.VIII.2008, coll. Han Huilin et al. (presented by Northeast Forestry University); 1♂, Dalian, 8–11.IX.2008, coll. Han Huilin et al. (presented by Northeast Forestry University); 5♂4♀, Faku, Chenwushitun; Wangyeling; Hongshadi; 23.VII–6.VIII.2006, coll. Wang Yiping (loaned from by insect specimen room of Nankai University); 6♂, Panjin, Rongxing, Youyan’gou, 18.IX.2017, coll. Wu Chunguang; 1♂, Rongxing, Xiaozhuangzi, 20.IX.2017, coll. Wu Chunguang. **Inner Mongolia** (IZCAS): 1♂, Yakeshi linqu, 20.VI.1983; 1♂, Yakeshi, Wuerhanqi, 30.VIII.1983; 1♂, Elunchunqi, Dayangshu, 370 m, 6.VII.1985, coll. Xue Dayong; 4♂1♀, Humeng, Arongqi, 23–25.VIII.1986, coll. Qi Shaofu et al.; 1♂, Zhemeng, Kulunqi, 20.VIII.1987, coll. Gong Bingwen. **Beijing** (IZCAS): 2♂, Beiping, 11.VII.1949, 8.VIII.1949; 11♂2♀, Xijiao Park, 6.V.1951, 10, 26–27.VIII.1951, 1–9.IX.1952, coll. Zhang Yiran et al.; 1♂, Qinghe, 30.VIII.1957, coll. Mao Jinlong; 7♂2♀, Sanpu, 23.VII–14.IX.1964, coll. Liao Subai et al.; 1♀, Mentougou, Liyuanling, 1100 m, 16.IX.2001, coll. Xue Dayong; 1♂, Chaoyang District, Institute of Zoology, Chinese Academy of Sciences, 24.VIII.2010, coll. Qi Feng; 3♂1♀, Miyun, 12.VII–11.IX.2010; 1♂, Changping, Liucun, Wangjiayuan, 160 m, 8.VIII.2008, coll. Wu Yupeng; 2♀, Changping, Baiyangcheng, 213 m, 15.VIII.2013, coll. Cui Le. **Hebei** (IZCAS): 1♀, Weixian, Xiheying, 9.IX.1964, coll. Han Yinheng; 1♀, Chicheng, Longmensuo, Liuzhuanzi, 10–11.VIII.2006, coll. Yang Chao. **Shandong** (IZCAS): 1♀, Lao Shan, 800 m; 5♂, Qihe, 13.VIII.1969. **Henan** (IZCAS): 6♂1♀, Xinxiang, 22.IV–14.IX.1973; 2♂, Xinyang, 26.VIII.1981, 26.VIII.1982; 1♂, Xinyang, Wuxingxiang, 230 m, 18.VII.2002, coll. Han Hongxiang; 1♂, Xinyang, Jigong Shan, 250 m, 20–21.VII.2002, coll. Han Hongxiang; 1♂, Xixia, Taiping, 834 m, 5–6.VIII.2013, coll. Jiang Nan et al. **Xinjiang** (IZCAS): 1♂, Zhaosu, Alasan, 2400 m, 24.VII.1978, coll. Han Yinheng; 1♀, Akesu, 1180 m, 18.VI.1978, coll. Han Yinheng; 3♂, Xinyuan, Yeguolin, 1280 m, 23–25.VII.2017, coll. Cheng Rui. **Shanghai** (IZCAS): 2♂1♀, 21.VI.1930, 19.VIII.1935, 17.IX.1935, coll. O. Piel. **Zhejiang** (IZCAS): 1♀, Wenzhou, 16.IX.1996, coll. Ding Jianqin. **Hubei** (IZCAS): 2♂1♀, Jinzhou, VII–VIII.1960; 1♀, Luotian, Qingtaiguan, 560 m, 1–4.VII.2014, coll. Jiang Nan. **Jiangxi** (IZCAS): 1♂, Guling, 10.VIII.1935, coll. O. Piel; 1♂, Wannian, X.1979. **Hunan** (IZCAS): 1♂, Hengyang, 24.VI.1981, coll. Li Jutao. **Yunnan** (IZCAS): 1♀, Jinping, Hetouzhai, 1700 m, 14.V.1956, coll. Huang Keren.

#### Distribution.

China (Heilongjiang, Jilin, Liaoning, Inner Mongolia, Beijing, Hebei, Shandong, Henan, Xinjiang, Shanghai, Zhejiang, Hubei, Jiangxi, Hunan, Yunnan), Russia (Ussuri), Korean Peninsula.

### Timandra apicirosea

Taxon classificationAnimaliaLepidopteraGeometridae

(Prout, 1935)

Figs 3 , 21 , 44 , 63

Calothysanis apicirosea Prout, 1935: 28, pl. 4: d. Holotype, Japan: Takao-San, near Tokyo (NHM).
Timandra apicirosea : Inoue 1977: 240.

#### Diagnosis.

This species is very similar to *T.distorta* based on the characters of the adult and the male genitalia. However, it can be distinguished from *T.distorta* by the combination of the following characters: the frons is deep reddish-brown in *T.apicirosea* but yellowish-brown without reddish pigmentation in *T.distorta*; in the male genitalia, the uncus is smaller in *T.apicirosea* than in *T.distorta*; the socii form a right angle and are without process at the base in *T.apicirosea*, while these are weakly curved and with a triangular process at the base in *T.distorta*; the processes on the dorsal and the ventral margin of the valvula are obviously longer in *T.apicirosea* than in *T.distorta*; the juxta is broader in *T.apicirosea* than in *T.distorta*. The female genitalia are similar to those of *T.dichela* in having a slender seventh sternite, but the posterior bifurcate parts are more extended outwards in *T.apicirosea*; the ductus bursae is shorter in *T.apicirosea* than in *T.dichela*; the sclerotized part of the ductus bursae is smaller in *T.apicirosea* than that in *T.dichela*.

#### Material examined.

**CHINA: Hubei** (IZCAS): 1♂, Yien, Changtanhe, Lianghekou, 949 m, 13–14.V.2017, coll. Li Henan. **Fujian** (IZCAS): 1♂, Shaxian, 25.VIII.1979, coll. Lin Naiquan; 1♀, Wuyi Shan, Sangang, 704 m, 21.X.2005, coll. Han Hongxiang; 1♂, Wuyi Shan, Taoyuanyu, 460 m, 24.X.2005, coll. Yang Chao. **Guangxi** (IZCAS): 1♀, Jinxiu, Luoxiang, 200 m, 15.V.1999, coll. Han Hongxiang; 1♀, Jinxiu, Zhong Gonglu, 1000 m, 10.V.1999, coll. Han Hongxiang; 2♀, Mao’er Shan, Gaozhai, 448 m, 13–15.VIII.2012, coll. Yang Chao et al.; 1♂, Mao’er Shan, Jiuniutang, 1146 m, 16.VIII.2012, coll. Yang Chao; 1♀, Huanjiang, Yangmei’ao, 1189 m, 18–22.VII.2015, coll. Li Xinxin. **Sichuan** (IZCAS): 1♀, Guanxian, Qingcheng Shan, 700–1600 m, 4.VI.1979, coll. Shang Jinwen; 2♂, Mianzhu, Jiulong Shan, Shizipo, 810 m, 29–31.VII.2016, coll. Cui Le; 1♂, Wenchuan, Sanjiang Fengjingqu, 1349 m, 25.VIII.2013, coll. Cheng Rui; 1♂, Hongya, Wawu Shan, Jinhuaqiao, 1147 m, 12–14.VIII.2016, coll. Cui Le.

#### Distribution.

China (Inner Mongolia, Gansu, Hubei, Jiangxi, Hunan, Fujian, Guangxi, Sichuan), Japan, Russia (Ussuri).

### Timandra distorta sp. n.

Taxon classificationAnimaliaLepidopteraGeometridae

http://zoobank.org/80704E88-40B0-4E5D-A001-7EB1243E9690

Figs 4 , 22 , 39 , 45

#### Description.

*Head.* Antennae bipectinate in basal four-fifths in male, dorsal surface of shaft yellowish-brown, sometimes coverded with brown scales, pectination covered with ciliae, basally black. Frons yellowish-brown with ventral side yellowish-white, slightly protruding (Fig. 39). Labial palpi yellowish-brown, reaching to tip of frons. Vertex pale yellowish-brown. *Thorax*. Patagia yellowish-brown. Tegulae and thorax pale yellowish-brown. Hind tibia with two pairs of spurs in male. Forewing length: male 12–15 mm. Forewing with apex pointed, slightly protruding; costa curved on terminal part; outer margin slightly protruding below vein M_3_. Apical angle of hindwing rounded, outer margin protruding on vein M_3_. Wing colour pale yellowish-brown, covered with blackish-brown spots especially on costal area of forewing. Forewing with discal spot reddish-brown and small; medial line reddish-brown, straight and narrow, arising from apex and extending to middle part of terminal margin; postmedial line overlapping with medial line near apex, separating from it on vein R_5_, slightly convex between veins M_3_ and CuA_1_; terminal line reddish-brown and very narrow. Fringes yellowish-brown, mixed with reddish-brown terminally. Hindwing with medial line reddish-brown, straight, narrow; postmedial line protruding between veins M_3_ and CuA_1_; terminal line and fringes similar to those of forewing. Underside densely covered with dark brown speckles; discal spot of forewing and postmedial lines of both wings darker than those of upperside.

***Male genitalia*.** Uncus small and stout, rounded at apex. Socii thin and flat, curved, covered with small spurs on surface, extending beyond tip of uncus; a short and triangular process present at base of outer margin of both socii. Costa with a ridge at approximately one-third from apex; valvula forming a narrow digitiform process both at apex of costa and on ventral margin, apical part of valvula between two processes membranous; sacculus short and stout, acute at tip. Juxta long, narrowing in terminal part. Aedeagus with basal half narrow; vesica partly sclerotized and crinkled.

***Female genitalia*.** Unknown.

#### Diagnosis.

The most discriminating character between *T.apicirosea* and *T.distorta* is the shape of the socii of the male genitalia. For more comparisons, see *T.apicirosea* above.

#### Type material.

Holotype, ♂, **CHINA: Tibet** (IZCAS): 1♂, Mêdog, Yarang, 1091 m, 20–23.VIII.2006, coll. Lang Songyun. Paratype: **Tibet** (IZCAS): 1♂, Bomi, 2750 m, 26.VIII.1982, coll. Han Yinheng.

#### Distribution.

China (Tibet).

#### Etymology.

The species is named referring to the Latin *distortus*, which refers to the curved socii in the male genitalia.

### Timandra dichela

Taxon classificationAnimaliaLepidopteraGeometridae

(Prout, 1935)

Figs 5 , 23 , 46 , 64

Calothysanis dichela Prout, 1935: 29, pl. 4: d. Syntypes ♂, Russia: S. Ussuri, Narva (NHM).
Timandra dichela : Inoue 1977: 240.

#### Diagnosis.

This species is very similar to *T.apicirosea* and *T.distorta* in its external characters. The species can be distinguished by the following characters: the colour of the frons is deeper in *T.dichela* than that in *T.apicirosea*, and without reddish pigmentation in *T.dichela*. In the male genitalia, the uncus is longer in *T.dichela* than in *T.apicirosea* and *T.distorta*; the socii are composed of two short acute processes on both sides of the tegumen in *T.dichela*, while these are much longer and digitiform in *T.apicirosea* and *T.distorta*; the process on the dorsal margin of the valvula is much stouter in *T.dichela* in comparison with *T.apicirosea* and *T.distorta*. The diagnostic characters of the female genitalia are given under *T.apicirosea*.

#### Material examined.

**RUSSIA** (NHM): 1♂, holotype, Narva, S Ussurigebie, 9.821, N Kardakoff, Joicey, Bequest, Brit. Mus. 1934-120. **CHINA: Henan** (IZCAS): 1♀, Xinyang, Jigong Shan, 250 m, 20–21.VII.2002, coll. Han Hongxiang. **Zhejiang** (IZCAS): 1♀, Yuyao, Siming Shan, 814 m, 31.VII–2.VIII.2016, coll. Li Xinxin; 1♂, Zhoushan, Putuo, Taohuodao, 40 m, 4.VIII.2016, coll. Li Xinxin. **Hubei** (IZCAS): 1♀, Shennongjia, Dajiuhu, 1800 m, 4.VIII.1981, coll. Han Yinheng; 1♂, Zigui, Maoping, 80 m, 27.IV.1994, coll. Yao Jian; 4♂1♀, Yingshan, Taohuachong, 590 m, 23–27.VI.2014, coll. Jiang Nan et al.; 4♂2♀, Yingshan, Wujia Shan, 860 m, 28–30.VI.2014, coll. Cui Le et al.; 1♂5♀, Luotian, Qingtaiguan, 560 m, 1–4.VII.2014, coll. Xue Dayong et al. **Jiangxi** (IZCAS): 1♂1♀, Jinggang Shan, Xiazhuang, 590 m, 5.VIII.2013, coll. Xue Dayong et al. **Hunan** (IZCAS): 1♂, Zhangjiajie, Wulingyuan, Wenfeng, 334 m, 11.V.2017, coll. Li Henan; 1♂, Yanling, Taoyuandong, 631 m, 4–8.VII.2008, coll. Chen Fuqiang; 2♂, Zhangjiajie, Wulingyuan, 267 m, 13.VI.2015, coll. Zhao Kaidong; 1♂, Zhangjiajie, Wulingyuan, Wenfeng, 350 m, 17.IX.2015, coll. Zhao Kaidong; 4♂6♀, Heng Shan, 22, 24, 29, 30.VIII.1979, 1–2.IX.1979, coll. Zhang Baolin; 1♀, Changsha, 29.VII.1983, coll. Zhang; 1♂, Zhangjiajie, 8.X.1988, coll. Fang Chenglai. **Fujian** (IZCAS): 3♂2♀, Wuyi Shan, Sangang, 704 m, 17.VIII.1979, 30.VI.1982, 20.X.2005, 11–14.VIII.2009, coll. Song Shimei et al.; 1♂, Linxia, 17.X.1980, coll. Huang Shuishi. **Guangdong** (IZCAS): 3♂, Shixing, Chebaling, 330 m, 1–2.VIII.2013, coll. Yang Chao et al.; 1♂, Ruyuan, Nanling conservation area, 1020 m, 16–20.VII.2008, coll. Chen Fuqiang. **Sichuan** (IZCAS): 1♀, Emei Shan, Qingyinge, 800–1000 m, 21.VI.1957, coll. Zhu Fuxing. **Yunnan** (IZCAS): 1♂, Yanjin, Hongli hotel, 469 m, 17–19.VIII.2016, coll. Cui Le.

#### Distribution.

China (Henan, Zhejiang, Hubei, Jiangxi, Hunan, Fujian, Taiwan, Hainan, Guangdong, Sichuan, Yunnan), SE Russia, Japan, Korean Peninsula, India.

### Timandra synthaca

Taxon classificationAnimaliaLepidopteraGeometridae

(Prout, 1938)

Figs 6 , 24 , 47

Calothysanis synthaca Prout, 1938: 154, pl. 16: g. Holotype ♂, Formosa [China: Taiwan] (central): Kagi district (NHM).
Timandra synthaca : Inoue 1992: 122.

#### Diagnosis.

This species is very similar to *T.apicirosea*. The following characters of the male genitalia distinguish it from *T.apicirosea*: the uncus is stouter; the socii are narrower and less strongly curved; the costa of the valva is more strongly sclerotized. The female genitalia of this species are unknown.

#### Material examined.

**CHINA: Taiwan** (NHM): 1♂, holotype, Central Formosa, Kagi district, Rothschild Bequest, 1939-1; **Taiwan** (BRCAS): 1♂, Hualien Co., Xinbaiyang (site 1), 1734 m, 22–23.VII.2015, coll. S. Wu.

#### Distribution.

China (Guangdong?, Taiwan).

#### Remarks.

Because the specimen in Wang (2011) was damaged (Min Wang pers. comm.), the record of the species from Guangdong is unconfirmed.

### Timandra convectaria

Taxon classificationAnimaliaLepidopteraGeometridae

Walker, 1861

Figs 7 , 25 , 41 , 48 , 65

Timandra convectaria Walker, 1861: 800. Holotype ♂, India: Bangladesh: Sylhet (NHM).
Calothysanis convectaria : Prout 1934: 56.

#### Diagnosis.

The medial line of the forewing arises from the inner side of the apex in *T.convectaria*, *T.correspondens*, *T.adunca*, and *T.quadrata*. However, *T.convectaria* is distinctive from *T.correspondens*, *T.adunca*, and *T.quadrata* as follows: a sharp protrusion is present on the frons of *T.convectaria*, *T.correspondens*, and *T.adunca*, while it is absent in *T.quadrata*; the middle part of the postmedial line of the hindwing is protruded outside in *T.convectaria* and *T.quadrata*, but it is straight in *T.correspondens* and *T.adunca*. In the male genitalia, *T.convectaria* and *T.quadrata* share the short process-like uncus and the flat apex of the valvula, while the uncus is raised and the apex of the valvula is rounded in *T.correspondens* and *T.adunca*; the arm between the valvula and the sacculus in *T.convectaria* is slightly shorter in the left side than in the right side, while in other three species, these arms are symmetrical, and more strongly curved in *T.adunca*; the costa of the valvula is broadened and protruding outwards in the basal half in *T.convectaria*, while it is strongly angled centrally in other three species; the cornutus is present as a narrow stripe in *T.convectaria*, but absent in *T.correspondens*, *T.adunca*, and *T.quadrata*. In the female genitalia, the seventh sternite is strongly sclerotized and divided into one large and one small sclerite in *T.convectaria*, but it is composed of a large sclerite with bifurcate apex on the posterior margin in *T.correspondens* and *T.adunca*. The ductus bursae of *T.convectaria* is much narrower than that of *T.correspondens* and *T.adunca*.

#### Material examined.

**INDIA** (NHM): 1♂, holotype, Sikim, N.W. India. **CHINA: Zhejiang** (IZCAS): 1♂, Jiangshan, Xingdun, 608 m, 10–12.VIII.2016, coll. Li Xinxin; 1♀, Zhoushan, Putuoqu, Taohuadao, 40 m, 2016.VIII.4, coll. Li Xinxin. **Hubei** (IZCAS): 1♀, Shennongjia, Jiuchong, 870 m, 19.VII.1998, coll. Ye Chanjuan. **Fujian** (IZCAS): 1♀, Nanjing, Tiankui, 6.XI.1980, coll. Zhang Baolin; 1♂, Wuping, Liangye Shan, Kongxia, 480–627 m, 17–19.XI.2008, coll. Chen Fuqiang. **Hainan** (IZCAS): 2♂, Baisha, Nankai, 270 m, 20–22.XI.2009, coll. Yang Chao. **Guangxi** (IZCAS): 1♂, Napo, Defu, 1350 m, 18.VI.2000, coll. Li Wenzhu et al.; 1♂, Longzhou, Nonggang conservation area, 195 m, 15–17.VII.2013, coll. Liu Shuxian; 1♀, Mao’er Shan, Gaozhai, 448 m, 13–15.VIII.2012, coll. Yang Chao; 2♂, Luchuan, Wenquan, Zhongxing, 198 m, 10.IV.2011, coll. Yang Xiushuai. **Sichuan** (IZCAS): 2♂, Emei Shan, Qingyinge, 800–1000 m, 25.IV.1957, coll. Wang Zongyuan et al. **Yunnan** (IZCAS): 1♂, Wanding, 900 m, 10.VI.1992, coll. Xue Dayong.

#### Distribution.

China (Zhejiang, Hubei, Hunan, Fujian, Hainan, Taiwan, Guangxi, Sichuan, Yunnan, Tibet), Russia, Japan, Korean Peninsula, India, Bangladesh, Vietnam, Philippines.

### Timandra correspondens

Taxon classificationAnimaliaLepidopteraGeometridae

Hampson, 1895

Figs 8 , 26 , 49 , 66

Timandra correspondens Hampson, 1895: 459. Syntypes, India: Dharmsala (NHM).
Calothysanis correspondens : Prout 1934: 56.

#### Diagnosis.

This species is very similar to *T.adunca* in the external characters: the postmedial line of the hindwing is without protrusion and straight; a sharp protrusion is present on the frons. Reliable identification of *T.correspondens* and *T.adunca* is possible using the genital characters: in the male genitalia, the arm between the valvula and the sacculus is less curved in *T.correspondens* than that in *T.adunca*, and reaches to the apex of the sacculus. The seventh sternite of the female *T.correspondens* is broader than that of *T.adunca*; the colliculum of *T.correspondens* is longer than that of *T.adunca*.

#### Material examined.

**CHINA: Tibet** (IZCAS): 1♀, Cona Xian, Mama, 2900 m, 6.VIII.1974, coll. Huang Fusheng; 1♀, Zayü, 2070 m, 2.VIII.1973; 1♂, Mêdog, Gutang, 2000 m, 3.X.1982, coll. Han Yinheng; 1♀, Mêdog, Baibung, 871 m, 17–18.VIII.2006, coll. Lang Songyun; 1♀, Mêdog, 1091 m, 22.VIII.2006, coll. Lang Songyun; 1♂, Zayü, Shang Zayü, 1960 m, 21–23.VIII.2005, coll. Wang Xuejian; 13♂2♀, Bomi, Tongmai, 2079–2100 m, 31.VIII.2005, 29–30.VIII.2006, coll. Wang Xuejian et al.

#### Distribution.

China (Yunnan, Tibet), India, Burma, Vietnam.

### Timandra adunca sp. n.

Taxon classificationAnimaliaLepidopteraGeometridae

http://zoobank.org/D54940B3-DF94-4F82-984A-6F3A92A150A6

Figs 9 , 27 , 50 , 67

**Description.***Head.* Antennae bipectinate in basal four-fifths in male, filiform in female; pectination covered with ciliae, black on basal part; dorsal surface of shaft deep brown with base reddish-brown. Frons yellowish-brown to deep yellowish-brown with ventral side yellowish-white, protruding with a sharp protrusion. Labial palpi yellowish-brown, sometimes reaching to tip of frons. Vertex pale yellowish-brown. *Thorax*. Patagia brown. Tegulae and thorax pale yellowish-brown. Fore leg with reddish-brown scales on dorsal side. Hind tibia with two pairs of spurs in male. Forewing length: male 14–17 mm, female 15–16 mm. Apex of forewing acute, slightly protruding; outer margin nearly straight; hindwing with sharped apical angle; outer margin angled on vein M_3_. Wing colour pale yellowish-brown covered with brown spots, costa of forewing dark brown with red suffusion. Forewing with deep reddish-brown and nearly triangular discal spot; medial line reddish-brown, straight and narrow, arising from inner side of apex and extending to middle part of terminal margin; postmedial line grey and narrow, separating from medial line on vein M_1_, slightly curved; fringes reddish-brown with blackish-brown at tip. Hindwing with medial and postmedial lines straight and narrow, the former reddish-brown and the latter grey; fringes similar to those of forewing. Underside with dark brown speckles; postmedial line on hindwing more distinct than on upperside.

***Male genitalia*.** Uncus raised. Socii present as a pair of narrow digitiform processes, beyond tip of uncus. Valvula with costa dilated at apex; arm between valvula and sacculus long and hook-like, extending beyond distal end of sacculus; sacculus very broad, rounded and densely covered with short setae terminally. Juxta long and narrow at terminal half. Saccus long and rounded at apex. Aedeagus slender and curved, without cornutus.

***Female genitalia*.** Seventh sternite trapezoid and strongly sclerotized with weakly bifurcate posterior margin. Colliculum short and narrow. Ductus bursae membranous with a membranous appendix bursae on posterior part, about two-fifths length of corpus bursae. Corpus bursae elongate oval; signum consists of a triangular pouch directed towards anterior part, with a longitudinal ridge running from apex.

#### Diagnosis.

See *T.correspondens*.

#### Type material.

Holotype, ♂, **CHINA: Yunnan** (IZCAS): Ruili, Wanting forest park, 900 m, 29.IV.2011, coll. Yang Xiushuai and Wang Ke. Paratypes: **Guangxi** (IZCAS): 2♂, Longsheng, Baiyan, 1150 m, 20.VI.1963, coll. Wang Chunguang; 1♀, Mao’er Shan, Antangping, 1579 m, 17–18.VIII.2012, coll. Yang Chao; 1♀, Huanjiang, Yangmeiao, 1189 m, 18–22.VII.2015, coll. Jiang Nan. **Guizhou** (IZCAS): 3♂, Panxian, Hongguozhen, Pengjiakou, 2065 m, 6–8.VII.2016, coll. Ban Xiaoshuang. **Yunnan** (IZCAS): 1♂, Lushui, Pianma, 1980 m, 3–4.VII.2014, coll. Pan Xiaodan; 1♂, Gong Shan, Dulongjiang, 1505 m, 8–9.VII.2014, coll. Pan Xiaodan; 1♂, Lushui, Pianma, 2300 m, 30.V.1981, coll. Liao Subai; 1♂, Yingjiang, 1700 m, 16.IV.1980, coll. Li Hongxing; 1♀, Tengchong, Heinitang, 1930 m, 28–30.V.1992, coll. Xue Dayong; 1♂, Tengchong, Dahaoping, 2020 m, 5–7.VIII.2007, coll. Wu Chunguang; 1♂, Tengchong, Qushi, Daba, 1823–1873 m, 4–5.VIII.2013, coll. Liu Shuxian; 1♂, Tengchong Shidi, 1730 m, 3–5.VIII.2016, coll. Ban Xiaoshuang; 4♂2♀, Tengchong, Houqiao, 1620 m, 6–8.VIII.2016, coll. Ban Xiaoshuang; 3♂, Pingbian, Dawei Shan, 1500–2090 m, 18.VI.1956, 19–20.VII.2016, 4–8.VIII.2017, coll. Huang Keren et al.; 1♂1♀, Wenshan, Malipo, Tianshengqiao, 1105 m, 7, 15.XI.2003, coll. Lu Shengxian. **Vietnam** (IZCAS): 1♀, 23.XI–2.XII.2012, coll. Chen Fuqiang.

#### Distribution.

China (Guangxi, Guizhou, Yunnan), Vietnam.

#### Etymology.

The species is named based on the Latin *aduncus*, which refers to the hook-like arm between the valvula and the sacculus in the male genitalia.

### Timandra quadrata sp. n.

Taxon classificationAnimaliaLepidopteraGeometridae

http://zoobank.org/18EDC70D-B76A-4AF5-85BD-A0CBD34D5307

Figs 10 , 28 , 51

#### Description.

*Head.* Antennae bipectinate in basal four-fifths in male, dorsal surface of shaft with reddish-brown scales, pectination yellowish-brown to blackish-brown. Frons dark yellowish-brown with ventral side yellowish-white and slightly protruding. Labial palpi yellowish-brown, not extending beyond frons. Vertex yellowish-white. *Thorax*. Patagia brown. Tegulae and thorax greyish-brown. Hind tibia with two pairs of spurs in male. Forewing length: male 14 mm. Apex of forewing pointed; outer margin weakly protruding; apex of hindwing rounded; outer margin forming a small protrusion on vein M_3_. Wing colour yellowish-brown. Forewing with costa covered with brown spots especially on basal half; antemedial line reddish-brown, very narrow and nearly straight; discal spot greyish-black and small, short bar-like; medial line reddish-brown; postmedial line greyish-brown, separated from medial line on vein M_1_, slightly protruding between M_2_ and CuA_1_; two blackish-brown spots present on veins R_4_ and R_5_ outside postmedial line; terminal line and fringes reddish-brown. Hindwing with medial line reddish-brown and straight; postmedial line grey, slightly protruding between M_3_ and CuA_1_; fringes similar to those of forewing. Underside densely covered with brown speckles; stripes deep brown; discal spot and postmedial line more distinct than those on upperside.

**Figures 28–35.**
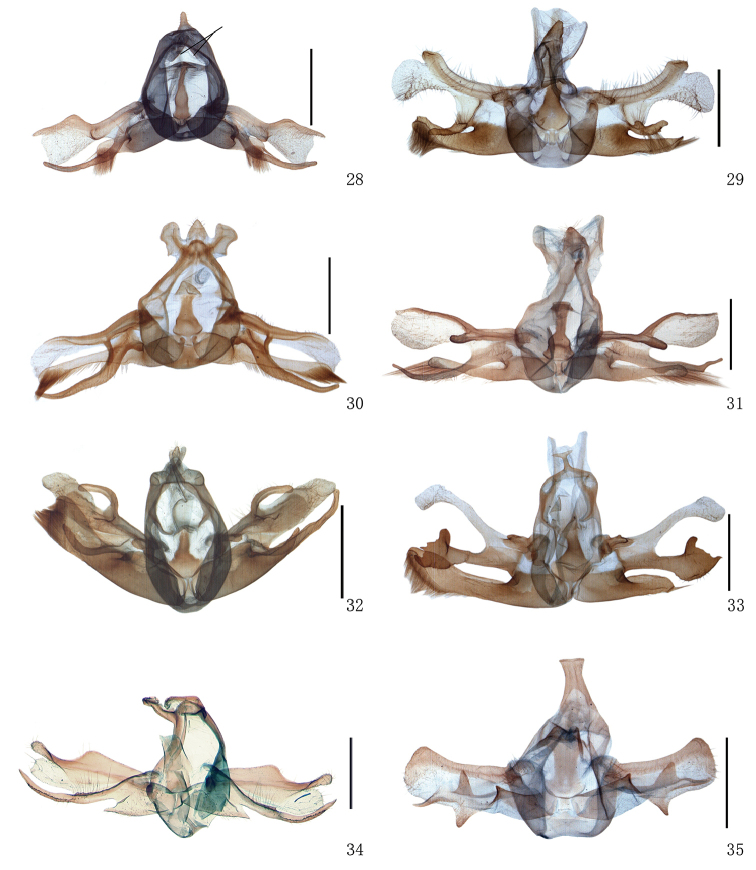
Male genitalia of *Timandra*. **28***T.quadrata* sp. n., black bars show the socii, Henan **29***T.accumulata* sp. n., holotype, Yunnan **30***T.comptaria*, Hubei **31***T.paralias*, Hebei **32***T.viminea* sp. n., holotype, Yunnan **33***T.oligoscia*, Yunnan **34***T.ruptilinea*, Guangdong **35***T.extremaria*, Hubei. Scale bar: 1 mm.

***Male genitalia*.** Uncus short, finger-like. Socii digitiform, short and broad, slightly beyond uncus. Costa broad at basal half, forming a right angle at terminal half, acute at apex; apex of valvula quadrate; a long, slightly curved digitiform arm stretching from cleft between valvula and sacculus; sacculus short, approximately half as long as valvula, narrow at apical one-third, rounded and setose at apex. Juxta long and narrow, tapered in terminal half. Saccus rounded at apex. Aedeagus slightly curved; vesica without cornutus.

***Female genitalia*.** Unknown.

#### Diagnosis.

*T.quadrata* is very closely related with *T.convectaria*, the medial line of the forewing is weakly curved, while it is absolutely straight in *T.convectaria*; a sharp protrusion is absent in *T.quadrata*, while it is present in *T.convectaria*; the postmedial line of the hindwing is less protruded in *T.quadrata* than in *T.convectaria*. The male genitalia of *T.quadrata* are also similar to those of *T.convectaria*, but the socii are slightly stouter than those of *T.convectaria*; the costa of the valvula is strongly angled centrally in *T.quadrata*, but straight in *T.convectaria*; the sacculus is much shorter in *T.quadrata* than in *T.convectaria*; the cornutus is absent in *T.quadrata*, but is present as a long stripe in *T.convectaria*. For more comparisions, see *T.convectaria* above.

#### Type material.

Holotype, ♂, **CHINA: Hubei** (IZCAS): 1♂, Shennongjia, Honghua, 860 m, 17.VIII.1981, coll. Han Yinheng. Paratype: **Henan** (IZCAS): 1♂, Baiyun Shan conservation area, 1550 m, 13–15.VIII.2008, coll. Xue Dayong.

#### Distribution.

China (Henan, Hubei).

#### Etymology.

The species is named based on the Latin *mancus*, which refers to the absence of the socii in the male genitalia.

### Timandra accumulata sp. n.

Taxon classificationAnimaliaLepidopteraGeometridae

http://zoobank.org/B5F05596-0DED-4340-AB87-26F28551E800

Figs 11 , 29 , 40 , 52 , 68

#### Description.

*Head*. Antennae bipectinate in basal five-sixths in male, filiform in female; dorsal surface of shaft pale yellowish-brown with brown scales except in basal part. Frons blackish-brown, mixed with yellowish-white on ventral side, forming a rounded protrusion (Fig. 40). Labial palpi yellowish-brown, not extending beyond frons. Vertex yellowish-white. *Thorax*. Patagia deep brown. Tegulae and thorax greyish-brown. Hind tibia with two pairs of spurs in male. Forewing length: male 16–18 mm. Forewing with acute apex; outer margin almost straight; apex of hindwing rounded; outer margin protruding on vein M_3_. Wing colour pale yellowish-brown densely scaled with greyish-brown spots. Forewing with indistinct antemedial line angled at cell; discal spot greyish-brown and short bar-like; medial line deep brown and oblique, raising from apex and extending to middle part of terminal margin, gradually broadening towards termen; postmedial line greyish-brown, overlapping with medial line at apex, separated from it before R_5_, slightly protruding; terminal line greyish-brown and narrow; fringes yellowish-brown. Hindwing with medial line deep brown and straight; postmedial line greyish-brown, slightly curved outwards in middle part; terminal line and fringes similar to those of forewing. Underside with deep greyish-brown spots, discal spot stronger and longer than upperside; postmedial line more distinct than that on upperside.

***Male genitalia*.** Uncus broad. Tegumen narrow. Socii straight, narrow and rod-like, slightly extending beyond tip of uncus. Costa of valva thickened and slightly curved at terminal half, quadrate apically; valvula forming a rounded protrusion and with bristles at apex, arm between valvula and sacculus short and digitiform with teeth on ventral margin, a small process present on base of costa, covered with bristles; sacculus slightly shorter than valvula and asymmetrical, right side slightly shorter than left side, apex of right side acute and rounded on left side; densely covered with long bristles terminally. Juxta with broad base, tapered towards terminal part. Saccus broad and rounded at apex. Aedeagus short, a long sclerotized band present on vesica.

***Female genitalia*.** Seventh sternite sclerotized, bifurcate at middle and forming two rounded lateral processes on posterior margin. Colliculum long. Ductus bursae short and membranous. Corpus bursae oval; signum present as a triangular pouch directed towards anterior part, with a longitudinal ridge running from apex.

#### Diagnosis.

The species is characterized by the very dense greyish-brown spots on the wings, which is similar to *T.rectistrigaria* (Eversmann, 1851), but the male and female genitalia of these two species are quite different (Hausmann 2004: fig. 177). The male genitalia are characterized by the following features: the uncus is stout; the socii are narrow and straight; the costa of the valvula is slightly curved, the arm between the valvula and the sacculus is short; the right side of the sacculus is shorter than the left side and acute apically; the left side of the sacculus is rounded apically. The female genitalia are similar to those of *T.recompta*, but the seventh sternite of *T.accumulata* is broader, the colliculum is longer in *T.accumulata*, and the appendix bursae of *T.accumulata* is absent.

#### Type material.

Holotype, ♂, **CHINA: Yunnan** (IZCAS): Lijiang, Yulong Shan, 10.VII.1962, coll. Song Shimei. Paratypes: 2♂, same as holotype, 21.VII.1962, coll. Song Shimei. **Yunnan** (ZFMK): 73♂13♀, Yunnan, Li-kiang, 6.VI–31.VIII.1934, 3–24.VII.1935, 12.VI.1935, 20.VI.1935, 21.IX.1935, 3.IX.1935, coll. H. Höne.

#### Distribution.

China (Yunnan).

#### Etymology.

The species is named based on the Latin *accumulatus*, which refers to the dense pattern of brown spots accumulated on the wing surface.

**Figures 36–42.**
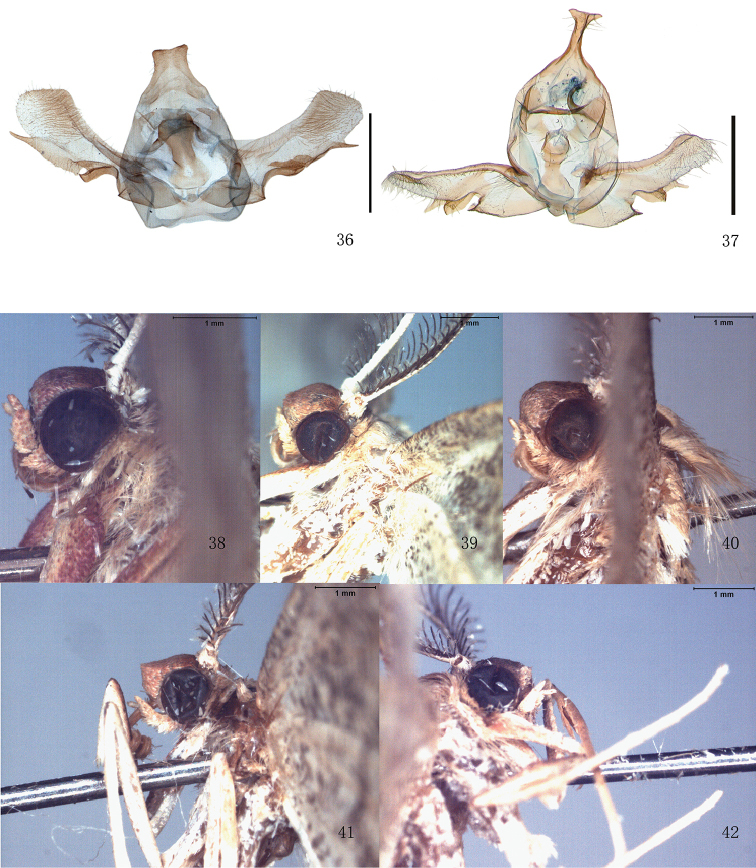
**36** Male genitalia, *T.robusta* sp. n., holotype, Yunnan **37** Male genitalia, *T.stueningi* sp. n., holotype, Taiwan **38–42** Frons of *Timandra*. **38***T.recompta***39***T.distorta* sp. n. **40***T.accumulata* sp. n. **41***T.convectaria***42***T.viminea* sp. n. Scale bar: 1 mm.

### Timandra comptaria

Taxon classificationAnimaliaLepidopteraGeometridae

Walker, 1863

Figs 12 , 30 , 53 , 69

Timandra comptaria Walker, 1863: 1615. Syntypes 1♂1♀, China; Hindostan [India] (NHM).
Timandra amata comptaria : Prout 1913: 48.
Calothysanis comptaria : Prout 1934: 55.

#### Diagnosis.

This species is similar to *T.paralias*, but the frons is less protruded than in *T.paralias* and the postmedial line of the hindwing is close to the medial line in *T.comptaria*, while it is far from the medial line in *T.paralias*. In the male genitalia, the arms between the valvula and the sacculus are symmetrical in *T.comptaria*, but these are asymmetrical in *T.paralias* and the right arm is slightly angled terminally; the sacculus is longer than the valvula in *T.comptaria*, while it is shorter than the valvula in *T.paralias*; the apex of the aedeagus is curved in *T.comptaria*, but it is straight in *T.paralias*; the cornutus is composed of one sclerotized stripe in *T.comptaria*, while in *T.paralias*, two sclerotized stripes are present on the vesica of *T.paralias*. The seventh sternite is slightly concave in the middle of the posterior margin in *T.comptaria*, but it is produced in *T.paralias*; in the female genitalia, the colliculum is absent in *T.comptaria*, while it is short funnel-shaped in *T.paralias* (Kaila and Albrecht 1994: fig. 17).

#### Material examined.

**CHINA: Taiwan** (NHM): 1♂, holotype, 1933/395; 1♀, 11.V.1906, A.E. Wileman, Rothschild Bequest, 1939-1; 1♂, Kanshirei, 1000 ft. 19.IV.1906, A.E. Wileman, Wileman Coll. B. M. 1929-261. **Heilongjiang** (IZCAS): 1♂2♀, Yichuan, 20–22.VI.1957, 26.VIII.1957; 26♂6♀, Yichuan, Dailing, 390 m, 24.VI–15.IX.1957, 13.VI–5.IX.1958, 26.VII.1959, 11.VIII.1959, 8.VI–6.VII.1962, coll. Bai Jiuwei et al.; 2♂1♀, Xiaoling, 30.VII.1938; 1♀, Wuchang, 7.VII.1970. **Jilin** (IZCAS): 3♂, Manjiang, 11, 17.VIII.1955. **Beijing** (IZCAS): 4♂6♀, Sanpu, 18–21.VIII.1964, 14.IX.1964, coll. Liao Subai; 1♂, Xiang Shan, 16.VIII.1957; 1♂, Yingtaogou, 29.VI.1990, coll. Zhao Jie; 1♀, Mentougou, Liyuanling, 1100 m, 11–12.VIII.2004, coll. Li Hongmei. **Hebei** (IZCAS): 1♂, Wuling Shan, 4.VIII.1981, coll. Gong Heng. **Shaanxi** (IZCAS): 2♂2♀, Fengxian, source of Jialingjiang, 1510 m, 21–24.VII.2017, coll. Cui Le; 1♂, Shangnan, gate of Jinsixia scenic area, 766 m, 16–19.VII.2017, coll. Cui Le. **Gansu** (IZCAS): 1♂, Yongdeng, Liancheng Linchang, 25.VI.1992, coll. Meng Feng; 1♂, Wenxian, VI–IX.2002, coll. Wang Hongjian. **Shanghai** (IZCAS): 5♂4♀, 15–27.VI.1933, 15–27.VIII.1933, 6.V.1935, coll. A. Savio. **Jiangsu** (IZCAS): 1♂, Yang Chow, 1936.V.15. **Zhejiang** (IZCAS): 1♀, Jiangshan, Xingdun, 608 m, 10–12.VIII.2016, coll. Li Xinxin; 2♂1♀, Yuyao, Siming Shan, 814 m, 31.VII–2.VIII.2016, coll. Li Xinxin; 1♂, Yinzhou, Chishui, 241 m, 25.VII.2015, coll. Cheng Rui; 2♂, Pan’an, Huangtan Linchang, 891 m, 27–28.VII.2015, coll. Cheng Rui; 1♂, Tianmu Shan, 20.VII.1973, coll. Zhang Baolin; 1♀, Jinyun, VII.1981; 1♂1♀, Qingyuan, Fengyang Shan, Datianping, 1290 m, 6–10.VIII.2003, coll. Han Hongxiang. **Hubei** (IZCAS): 1♂5♀ (Shennongjia, Dajiuhu, 1800 m, 1–5.VIII.1981, coll. Han Yinheng. **Jiangxi** (IZCAS): 2♀, Guling, VII.1935, 19.VIII.1935; 1♀, Lu Shan, 17.VI.1974, coll. Zhang Baolin; 1♀, Jiulian Shan, 23.VI.1975, coll. Song Shimei. **Hunan** (IZCAS): 1♂1♀, Anhua, 25.VIII.1981; 4♂, Hengyang, Nanyue Linchang, 13.VII.1980, 6–28.IX.1980, coll. Li Jutao et al.; 1♂1♀, Mang Shan, 30.VI.1981, 13.VII.1981. **Fujian** (IZCAS): 1♂1♀, Nanping, Xiadao, 27–28.V.1963, coll. Zhang Youwei; 2♂1♀, Jianyang, Huangkeng, 270 m, 30.VI–1.VII.1973, coll. Zhang Bailin; 1♂, Jianyang, Huangkeng, Aotou, 25.VI.1980, coll. Jiang Fan; 1♂, Jiangyang, Chengguan, 90–120 m, 12.VIII.1960, coll. Ma Chenglin; 1♀, Jianou, Dongfeng, 27.X.1980; 1♂, Wuyi Shan, 25.IV.1982, coll. Zhang Baolin; 1♀, Wuyi Shan, Sangang, 704 m, 11–14.VIII.2009, coll. Xue Dayong; 1♂, Meihua Shan, Yunshan, 459 m, 18.VII.2013, coll. Yang Chao. **Guangdong** (IZCAS): 1♀, Shixing, Chebaling, 330 m, 1–2.VIII.2013, coll. Xue Dayong. **Sichuan** (IZCAS): 10♂10♀, Emei Shan, Qingyinge, 800–1000 m, 16.IV–15.VII.1957, 17–22.IX.1957, coll. Zhu Fuxing et al.; 1♂, Emei Shan, Jiulaodong, 1800–1900 m, 14.VIII.1957, coll. Zhu Fuxing; 1♂, Guanxian, Qingcheng Shan, 700–1600 m, 30.V.1979, coll. Gao Ping; 1♀, Wanxian, Wang’erpu, 1200 m, 27.IX.1994, coll. Song Shimei. **Chongqing** (IZCAS): 1♀, 14.VI.1974, coll. Han Yinheng; 1♀, Beibei, 14.V.1999, coll. Wang Haijian et al. **Yunnan** (IZCAS): 1♀, Jingdong, 1170 m, 2.VI.1956, coll. A.K. Zaguljaev; 1♂, Menglong, Bannan, Mengsong, 1600 m, 24.IV.1958, coll. Meng Xuwu; 1♀, Zhenxiong, Machang, 1820 m, 24.VII.1982, coll. Luo Zhengjin; 5♂3♀, Menglong, Bannan, Mengso,1930 m, 28–30.V.1992, coll. Xue Dayong.

#### Distribution.

China (Heilongjiang, Jilin, Beijing, Hebei, Shaanxi, Gansu, Shanghai, Jiangsu, Zhejiang, Hubei, Jiangxi, Hunan, Fujian, Taiwan, Guangdong, Sichuan, Chongqing, Yunnan), Russia, Korean Peninsula, Japan, India.

### Timandra paralias

Taxon classificationAnimaliaLepidopteraGeometridae

(Prout, 1935)

Figs 13 , 31 , 54

Calothysanis paralias Prout, 1935: 28, pl. 4, fig. c. Holotype ♂, Russia: Siberia (east), Vladimir Bay, at mouth of river (NHM).
Timandra paralias : Kaila and Albrecht 1994: 461.

#### Diagnosis.

In the male genitalia, *T.paralias* is unique by the angled apex of the right arm between the valvula and the sacculus. For more detailed comparisons with *T.comptaria*, see *T.comptaria*.

#### Material examined.

**CHINA: Heilongjiang** (IZCAS): 3♂, Yichun, Dailing, 390 m, 15.VII.1958, 26.VII.1959, 11.VIII.1959, coll. Zhou Shixiu et al. **Inner Mongolia** (IZCAS): 1♂, Jiwen, 16.VII.1982; 2♂, Chifeng, Eqi, 12–14.VII.1987, coll. Liu Chunxiang et al. **Hebei** (IZCAS): 1♂, Xiaowutai Shan, Nantai, 1700 m, 7.VII.1964, coll. Wang Chunguang; 1♂, Chicheng, Longmensuo, Liuzhuangzi, 10–11.VIII.2006, coll. Yang Chao.

#### Distribution.

China (Heilongjiang, Inner Mongolia, Hebei), Russia (Siberia).

**Figures 43–60.**
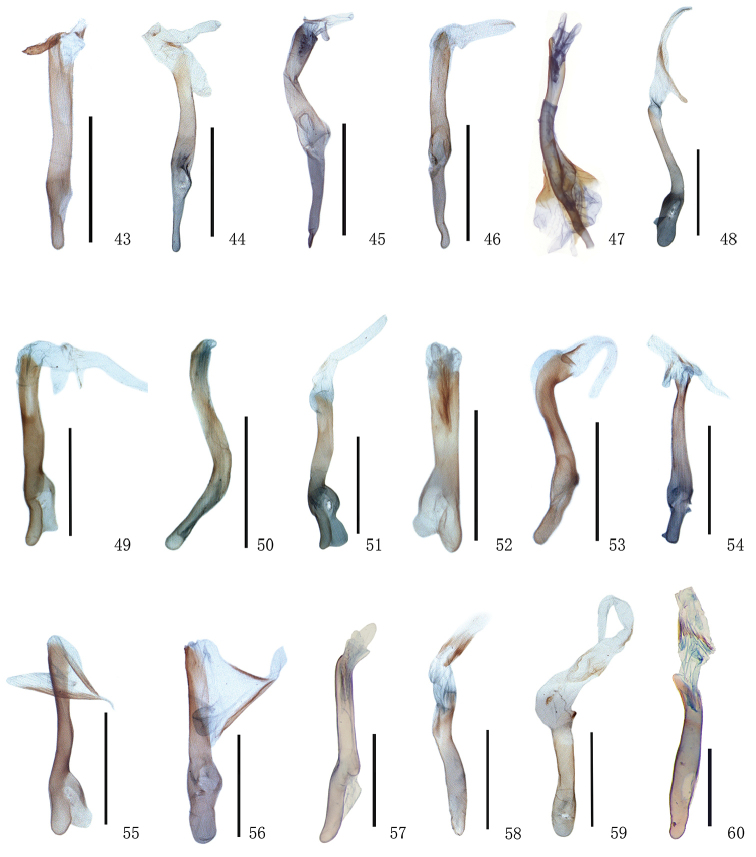
Aedeagus of *Timandra*. **43***T.recompta*, Gansu **44***T.apicirosea*, Sichuan **45***T.distorta* sp. n., holotype, Tibet **46***T.dichela*, Hubei **47***T.synthaca*, Taiwan **48***T.convectaria*, Hainan **49***T.correspondens*, Tibet **50***T.adunca* sp. n., holotype, Guangxi **51***T.quadrata* sp. n., Henan **52***T.accumulata* sp. n., holotype, Yunnan **53***T.comptaria*, Hubei **54***T.paralias*, Heilongjian **55***T.viminea* sp. n., holotype, Yunnan **56***T.oligoscia*, Yunnan **57***T.ruptilinea*, Guangdong **58***T.extremaria*, Hubei **59***T.robusta* sp. n., holotype, Yunnan **60***T.stueningi* sp. n., holotype, Taiwan. Scale bar: 1 mm.

### Timandra viminea sp. n.

Taxon classificationAnimaliaLepidopteraGeometridae

http://zoobank.org/9942C321-7E9D-4FDA-96CA-597FE443E659

Figs 14 , 32 , 42 , 55 , 70

#### Description.

*Head*. Antennae bipectinate to five-sixths in male and filiform in female; dorsal surface of shaft pale yellowish-brown, slightly speckled with brown scales. Frons blackish-brown, ventral side yellowish-white and with an obtuse protrusion on ventral margin (Fig. 42). Labial palpi yellowish-brown, not extending beyond frons. Vertex yellowish-white. *Thorax*. Patagia brown. Tegulae and thorax pale yellowish-brown. Hind tibia with two pairs of spurs in male. Forewing length: male and female 12–13 mm. Apex of forewing acute and outer margin nearly straight; hindwing with rounded apex and outer margin forming a small protrusion on vein M_3_. Wing colour yellowish-brown with brown spots. Forewing with discal spot blackish-brown, small, angled at middle; medial line reddish-brown, arising from apex; postmedial line grey and narrow, separating from medial line on vein R_5_; terminal line grey; fringes yellowish-brown, sometimes pinkish-red at apex. Hindwing with medial line reddish-brown and straight; postmedial line grey and narrow, protruding centrally; terminal line and fringes similar to those of forewing. Underside densely covered with brown speckles; postmedial line more distinct than that on upperside.

***Male genitalia*.** Uncus broad at base, digitiform at terminal part. Socii narrow and rod-like, extending slightly beyond tip of uncus; inner margin of tegumen with a pair of acute processes centrally. Costa of valva with a digitiform and curved process centrally; a slender and curved arm present between valvula and sacculus; sacculi asymmetric, terminal part broad and narrow, covered with setae on left side, but narrow and digitiform on right side. Juxta broad near base, tapered towards terminal part. Saccus small and rounded at apex. Aedeagus slender, cornuti composed of two long sclerotized stripes.

***Female genitalia*.** Seventh sternite narrow and sclerotized, slightly concave on posterior margin. Lamella postvaginalis sclerotized. Colliculum narrow. Ductus bursae shorter than corpus bursae, strongly sclerotized on posterior part; signum with a longitudinal sclerite inwards a slightly sclerotized plate, a pouch present on anterior part.

#### Diagnosis.

The new species can be identified by the combination of the following characters: the frons is blackish-brown with an obtuse protrusion on the ventral margin; in the male genitalia, a long digitiform and curved process is present at the middle of the costa; the sacculi are asymmetric, as the left side is much stouter than the right one; the cornuti are two long sclerotized stripes; the seventh sternite of the female is narrow and sclerotized, and slightly concave on the posterior margin; the posterior half of the ductus bursae is strongly sclerotized.

#### Type material.

Holotype, ♂, **CHINA: Yunnan** (IZCAS): Baoshan, Baihualing, 1520 m, 11–13.VIII.2007, coll. Xue Dayong. Paratypes: 3♂7♀, as same as holotype, coll. Wu Chunguang et al.; 3♂, Cang Shan, Puladi, 1298 m, 6–7.VII.2014, coll. Li Xinxin; 1♀, Baoshan, Bawan, 1040 m, 8–10.VIII.2007, coll. Xue Dayong.

#### Distribution.

China (Yunnan).

#### Etymology.

The species is named based on the Latin *vimineus*, which refers to the slender right sacculus in the male genitalia.

### Timandra oligoscia

Taxon classificationAnimaliaLepidopteraGeometridae

Prout, 1918

Figs 15 , 33 , 56 , 71

Timandra oligoscia Prout, 1918: 79. Syntypes ♂, China: Tibet, Vrianatong (NHM).
Calothysanis oligoscia : Prout 1934: 56.

#### Diagnosis.

This species can be distinguished from its congeners by the following characters: the frons is blackish-brown and slightly protruded; the postmedial line of the forewing is almost straight and separating from the medial line under the vein M_3_. In the male genitalia, the uncus is narrow and T shaped; the socii are long and extending beyond the tip of the uncus; the terminal part of the valvula is weakly sclerotized, its apex is slightly dilated and almost rectangular; the arm between the valvula and the sacculus is as long as the valvula and strongly sclerotized, its apex is dilated, plate-like with irregular serration on the apical margin; both sacculi have a rounded apex, but are asymmetric, as the left side is longer than the right one; the posterior margin of the aedeagus has several small teeth; the cornuti are developed as two sclerotized stripes. The seventh sternite of the female is large, strongly sclerotized, connected with the tergum as a ring, and forms two diverticula on the anterior margin; the colliculum in the female genitalia is stout; the ductus bursae is short and stout.

#### Material examined.

**CHINA: Gansu** (IZCAS): 1♀, Zhouqu, Shatan Linchang, 2400 m, 15.VII.1999, coll. He Tongli. **Hubei** (IZCAS): 1♀, Shennongjia, Dajiuhu, 1800 m, 5.VIII.1981, coll. Han Yinheng. **Hunan** (IZCAS): 1♀, Zhangjiajie, Wulingyuan, Huanglonglu, 348 m, 18.IX.2015, coll. Zhao Kaidong. **Guangxi** (IZCAS): 1♂2♀, Napo, Defu, 1300–1350 m, 16.VIII.1998, 18.VI.2000, coll. Li Wenzhu et al. **Sichuan** (IZCAS): 1♀, Dukou, 22.VIII.1980, coll. Zhang Baolin; 1♂, Huili, 24.VII.1974, coll. Han Yinheng. **Yunnan** (IZCAS): 1♂2♀, Xinping, Gasa, Yaonan, 1900 m, 10–13.VIII.2017, coll. Cui Le; 1♂, Tengchong Shidi, 1697 m, 25.VI.2014, coll. Li Xinxin; 1♂, Weixi, Pantiange, 2570 m, 15–16.VII.2014, coll. Pan Xiaodan; 1♂, Dali, Cang Shan, 2226 m, 23–24.VI.2014, coll. Li Xinxin; 1♂, Changning, 25.VI.1979; 4♂, Dali, Hudiequan, 2050 m, 4.VI.1980, 16.V.1992, coll. Xue Dayong; 1♂, Dali, Zhonghe (Dali, Cang Shan), 2120 m, 12–13.VIII.2013, coll. Li Xinxin; 1♂, Tengchong, Laifeng Shan, 1620 m, 24.V.1982; 1♂, Tengchong, Qushi, Daba, 1823–1873 m, 4–5.VIII.2013, coll. Liu Shuxian. **Tibet** (IZCAS): 1♂, Zayü, Shang Zayü, 1963 m, 30.VI–1.VII.2015, coll. Li Xinxin; 2♂, Zayü, Shang Zayü, 1960 m, 21–23.VIII.2005, coll. Wang Xuejian.

#### Distribution.

China (Gansu, Hubei, Hunan, Guangxi, Sichuan, Yunnan, Tibet), Burma.

### Timandra ruptilinea

Taxon classificationAnimaliaLepidopteraGeometridae

Warren, 1897

Figs 16 , 34 , 57 , 72

Timandra ruptilinea Warren, 1897: 64. Holotype ♂, India: Khasi Hills (NHM).
Timandra flavisponsaria Hampson, 1912: 1248. Syntypes, India: Madras, Wynâd; Nilgiris; Burma, Katha (NHM).

#### Diagnosis.

This species differs from its congeners by the following characters: the wing colour of the area outside the medial line is darker than the area inside the medial line; the postmedial line of the forewing is narrow and forms black spots on the veins; the middle part of the postmedial line of the hindwing is strongly curved. In the male genitalia, the uncus is small and raised in *T.ruptilinea* , which is similar to that of *T.correspondens* and *T.adunca*; the socii are long and digitiform, extending beyond the tip of the uncus; the costa of the valva is narrow at the terminal half and rounded at the tip; the arm between the valvula and the sacculus is longer than the valvula and as long as the costa of the valva, and equipped with several small teeth on the ventral margin, except on the basal half and the subapical part; the sacculus is short and acute at the apex; the vesica of the aedeagus is weakly sclerotized and wrinkled. The seventh sternite of the female is short and bifurcated on the posterior margin; the lamella postvaginalis in the female genitalia are three quadrate lobes, the central one is less sclerotized than the lateral ones; the ductus bursae is very short, narrow, and sclerotized posteriorly.

#### Material examined.

**CHINA: Guangdong** (loaned from ZFMK): 1♂, China mer.occ. Kwangtung sept, Lienping, ex coll. Wehrli. **Guangxi** (ZFMK): 2♂1♀, China (Kouangsi), Région da Nanning, 1929.

#### Distribution.

China (Guangdong, Guangxi), India, Burma, Malay Peninsula.

### Timandra extremaria

Taxon classificationAnimaliaLepidopteraGeometridae

Walker, 1861

Figs 17 , 35 , 58 , 73

Timandra extremaria Walker, 1861: 801. Holotype ♂, N China (NHM).
Timandra ? sordidaria Walker, 1863: 1615 (N China).
Calothysanis extremaria : Prout 1934: 57.
Calothysanis extremaria xenophyes Prout, 1935: 29, pl. 4, fig. c.

#### Diagnosis.

This species, *T.robusta* and *T.stueningi* differ from its congeners by the following characters: the frons is fully protruded; the postmedial lines of both two wings form black spots on the veins. In the male genitalia, the tip of the uncus is flat and the socii are absent. *T.extremaria* can be distinguished from *T.robusta* and *T.stueningi* by the following characters: the forewing is shorter and broader in *T.stueningi* than in *T.extremaria* and *T.robusta*; the outer margin of the forewing is straighter in *T.stueningi* than in *T.extremaria* and *T.robusta*; the colour of the frons is blackish-brown with reddish pigmentation in *T.extremaria*, but without reddish pigmentation in *T.robusta*. In the male genitalia, the uncus of *T.robusta* is shorter and stouter than that of *T.extremaria* and *T.stueningi*, the basal half in *T.stueningi* is narrower than in *T.extremaria* and *T.robusta*; a pair of spurs are present on the inner side of the tegumen in *T.robusta* and *T.stueningi*, while they are absent in *T.extremaria*, but in *T.stueningi*, the spurs are longer than those in *T.robusta*; a triangular process is present at the central part of the valvula in *T.extremaria*, but it is absent in *T.robusta* and *T.stueningi*; the apex of the sacculus is more acute in *T.extremaria* and *T.stueningi* than in *T.robusta*; the middle process on the ventral margin of the valvula is triangular in *T.extremaria*, but digitiform in *T.robusta* and *T.stueningi*; the vesica is more strongly sclerotized in *T.extremaria* and *T.stueningi* than in *T.robusta*. In the female genitalia, the ductus bursae of *T.extremaria* is broader than that of *T.stueningi*.

#### Material examined.

**CHINA**: 1♂, holotype of *T.extremaria*, N. China, 54.8 (NHM). 1♂, holotype of *T.sordidaria*, N. China (NHM). **Shaanxi** (IZCAS): 1♀, Ningshan, Huoditang, 4.VIII.1979, coll. Han Yinheng; 1♂, Ningshan, Guanghujie conservation area 1189 m, 26–28.VII.2014, coll. Ban Xiaoshuang; 1♀, Ziyang, 21.VI.1976, coll. Ma Wenzhen. **Gansu** (IZCAS): 2♂, Wenxian, VI–IX.2002, coll. Wang Hongjian; 1♀, Wenxian, Fanba, 800 m, 26.VI.1998, coll. Zhang Xuezhong; 1♀, Wenxian, Bikou, 620 m, 15–16.VIII.2014, coll. Ban Xiaoshuang; 2♂, Wenxian, VI–IX.2002, coll. Wang Hongjian; 1♂, Bikou, Bifenggou, 720 m, 8–10.VIII.2016, coll. Cheng Rui and Jiang Shan. **Shanghai** (IZCAS): 8♂3♀, 15.VI.1930, 21.VI.1933, 5–23.VIII.1933, 7.VI.1934, 17, 19.V. 1935, coll. O. Piel and A. Savio. **Anhui** (IZCAS): 1♀, Sucheng, 9.VIII.1955. **Zhejiang** (IZCAS): 3♂, Lin’an, West Tianmu Shan, 400 m, 2003.VII.26–27, coll. Xue Dayong; 1♀, Hangzhou, 20.X.1980, coll. Zhang Bailin; 1♂, Tianmu Shan, Longyuan Shanzhuang, 10.V.1998, coll. Xia Weizheng; 10♂3♀, West Tianmu Shan, 30.VII–1.VIII.1972, 20–24.VII.1973, 17.X.1980, coll. Zhang Bailin et al.; 4♂, Lin’an, West Tianmu Shan, 400 m, 26–27.VII.2003, coll. Xue Dayong; 1♀, Chekiang, Chusan (Zhoushan), 27.VII.1931, coll. O. Piel; 3♂, Tianmu Shan, 7–10.VII.2007, coll. You Ping (loaned from insect specimen room of Nankai University). **Hubei** (IZCAS): 4♂, Yunxi, Guanyinzhen, 289–305 m, 4–5.VIII.2014, coll. Liu Shuxian; 1♂1♀, Xingshan, Longmenhe, 730–1260 m, 22–23.VI.1993, coll. Huang Runzhi et al.; 1♂, Xingshan, Xiaohekou, 700 m, 11.V.1994, coll. Li Wenzhu. **Hunan** (IZCAS): 1♂, Heng Shan, 1980; 1♂1♀, Hengyang, Nanyue Linchang, 28.VI.1980, 3.X.1980, coll. Li Jutao et al.; 1♂, Nanyue, Shumuyuan, 12.VII.1980, coll. Li Jutao; 1♂1♀, Hengyang, Nantaisi, 24–25.VIII.1980, coll. Liu Yili et al.; 1♂, Zhangjiajie, 11.X.1988; 1♂, Guzhang, Gaowangjie, 850 m, 29.VII.1988, coll. Chen Yixin; 1♂2♀, Heng Shan, 21–29.VIII.1979, coll. Zhang Baolin; 1♀, Fenghuang Xiancheng, 362 m, 25.IX.2015, coll. Yao Jian; 1♀, Fenghuang, Nanhua Shan, 456 m, 26.IX.2015, coll. Zhao Kaidong; 1♀, Shimen, Huping Shan, Nanping, Maozhuhe, 320 m, 15–17.X.2014, coll. Yao Jian; 1♀, Shimen, Huping Shan, Daling, 444 m, 18–20.X.2014, coll. Yao Jian. **Fujian** (IZCAS): 1♂, Jianou, Dongfeng, 1.II.1980; 2♀, Wuyi Shan, 4–5.VII.1982, coll. Zhang Kechi et al. **Taiwan** (NHM): 1♂, Kanshirei, Formosa, 1000 ft. 2.VIII.1906, A.E. Wileman, Wileman Coll. B. M. 1929-261. 1♀, Kanshirei, Formosa, 1000 ft. 20.IV.1906, A.E. Wileman, Wileman Coll. B. M. 1929-261. **Guangxi** (IZCAS): 2♂, Ziyuan, 14.VII.1976, coll. Zhang Baolin; 1♂1♀, Longsheng, 13.VI.1980, coll. Song Shimei; 1♂, Napo, Defu, 1350 m, 19.VI.2000. **Sichuan** (IZCAS): 28♂15♀, Emei Shan, Qingiynge, 800–1000 m, 29.IV–26.VII.1957, 17–22.IX.1957, coll. Zhu Fuxing et al.; 1♀, Emei Shan, Baoguosi, 550–750 m, 24.IV.1957, coll. Wang Zongyuan; 5♂1♀, Emei Shan, 580–1100 m, 20–21.VI.1955, 11.VI.1974, 19.VI.1979, 29.VIII.1980, coll. Li Jinhua et al.; 1♀, Lu Shan, 31.VII.1980, coll. Zhang Baolin; 1♂1♀, Huili, 1900 m, 10.X.1960, 31.VII.1974, coll. Meng Xuwu et al.; 2♂, Guanxian, Qingcheng Shan, 700–1600 m, 23.VI.1963, 4.VI.1979, coll. Zhang Xuezhong et al.; 2♂2♀, Xichang, 29–31.VII.1980, coll. Zhang Baolin. **Guizhou** (IZCAS): 1♂, Leishan, Leigong Shan, 1650 m, 2.VII.1988, coll. Yuan Dechang.

#### Distribution.

China (Shaanxi, Gansu, Shanghai, Anhui, Zhejiang, Hubei, Hunan, Fujian, Taiwan, Guangxi, Sichuan, Guizhou).

**Figures 61–68.**
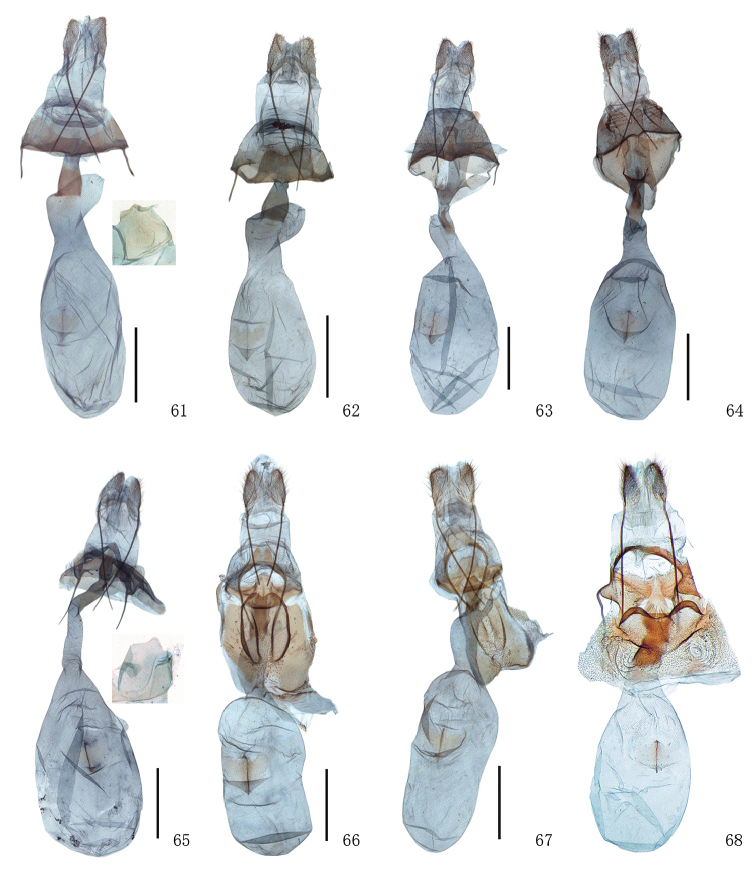
Female genitalia of *Timandra*. **61***T.griseata*, Xinjiang **62***T.recompta*, Xinjiang **63***T.apicirosea*, Sichuan **64***T.dichela*, Jiangxi **65***T.convectaria*, Fujian **66***T.correspondens*, Tibet **67***T.adunca* sp. n., Yunnan **68***T.accumulata* sp. n., Yunnan. Scale bar: 1 mm.

### Timandra robusta sp. n.

Taxon classificationAnimaliaLepidopteraGeometridae

http://zoobank.org/5D00CE17-814D-4D32-92AB-CD98AEE1DA01

Figs 18 , 36 , 59

#### Description.

*Head*. Antennae bipectinate in basal four-fifths in male; dorsal surface of shaft pale yellowish-brown with brown scales to tip gradually. Frons deep yellowish-brown and slightly protruding. Labial palpi yellowish-brown, not extending beyond frons. Vertex yellowish-white, sometimes mixed with brown centrally. *Thorax*. Patagia brown. Tegulae and thorax greyish-brown. Hind tibia with two pairs of spurs in male. Forewing length: male 18–19 mm. Forewing with pointed apex; outer margin almost straight; hindwing with rounded apex; outer margin protruding on vein M_3_. Wing colour yellowish-brown. Forewing with discal spot black and weak; medial line brown, straight, arising from apex and extending to middle part of terminal margin; postmedial line grey, straight, narrow and weak, forming a row of small black spots on veins, separating from medial line before vein M_1_; terminal line brown; fringes yellowish-brown. Hindwing with medial line brown, straight; postmedial line arched, similar to that of forewing; terminal line and fringes similar to those of forewing. Underside with terminal line of forewing, postmedial line of hindwing and discal spot of forewing more distinct than those on upperside.

***Male genitalia*.** Uncus short and stout, slightly concave at tip. Socii absent. A pair of short spurs present on inner side of tegumen. Base of ventral margin of valvula with two short processes, basal one stouter than subapical one. Sacculus short with acute apex. Juxta broad on basal and terminal part, narrow centrally. Saccus short and broad, terminally flattened. Aedeagus short and narrow; vesica membranous, but partly weakly sclerotized, with two small sclerotized protrusions, covered with spurs on surface.

***Female genitalia*.** Unknown.

#### Diagnosis.

See the diagnosis of *T.extremaria*.

#### Type material.

Holotype, ♂, **CHINA: Yunnan** (IZCAS): Baoshan, Baihualing, 1520 m, 11–13.VIII.2007, coll. Wu Chunguang. Paratype: **Yunnan** (IZCAS): 1♂, Jingdong, 1170 m, 1.VI.1956, coll. A.K. Zaguljaev.

#### Distribution.

China (Yunnan).

#### Etymology.

The species is named based on the Latin *robustus*, which refers to the short and stout uncus in the male genitalia.

### Timandra stueningi sp. n.

Taxon classificationAnimaliaLepidopteraGeometridae

http://zoobank.org/872AB617-27B0-4912-9204-50C1BDD94EF8

Figs 19 , 37 , 60 , 74

#### Description.

*Head*. Antennae bipectinate in male; dorsal surface of shaft pale yellowish-brown with brown scales, except in basal part; filiform in female. Frons reddish-brown, scaled with yellowish-white on ventral side, not protruding. Labial palpi yellowish-brown, slightly extending beyond frons. Vertex yellowish-white. *Thorax*. Patagia greyish-brown. Tegulae and thorax greyish-brown. Hind tibia with two pairs of spurs in male. Forewing length: male and female 17–19 mm. Forewing with acute apex, slightly protruded, outer margin straight; hindwing with rounded apex, outer margin protruding on vein M_3_. Wing colour yellowish-brown, densely covered with blackish or greyish-brown spots. Forewing with discal spot nearly invisible, greyish-brown; medial line arising from apex, narrow and black apically, then reddish-brown and straight, broad below vein R_5_; postmedial line composed of black spots on veins and connected with greyish-brown thin line; terminal line pale brown; fringes yellowish-brown. Hindwing with medial line similar to that of forewing; postmedial line arched, similar to that of forewing; terminal line and fringes similar to those of forewing. Underside densely covered with black speckles, stripes blackish-brown. Postmedial line of all wings and discal spot of forewing more distinct than those on upperside.

***Male genitalia*.** Uncus narrow, dilated at terminal part, resembling fishtail. Socii absent. Tegumen with a pair of long and curved spine-like processes on inner side. Costa narrow; valvula rounded at apex, with two short processes centrally, basal one stout and rounded at tip, apical one narrow and acute terminally; sacculus approximately two times shorter than valvula, acute at tip. Juxta rounded at base, middle part slightly narrower than terminal part. Saccus small. Aedeagus slightly curved terminally; vesica with a weakly sclerotized and ribbed band.

***Female genitalia*.** Seventh sternite membranous. Sterigma large, widely concave at middle of posterior margin. Colliculum short. Ductus bursae slender, approximately half as long as corpus bursae. Corpus bursae long and oval; signum with a longitudinal sclerite inside a slightly sclerotized plate, a pouch present on anterior part.

#### Diagnosis.

See *T.extremaria*.

#### Type material.

**CHINA: Taiwan**: Holotype (ZFMK), ♂, Chilan (Ilan Cy), 600 m, 16.VI.1993, coll. F. Aulombard and J. Plante. Paratypes: **Taiwan**: 1♂, Tibn hsiang, Hualien Co., 600 m, 19/21.VI.1993, coll. F. Aulombard and J. Plante (loaned from ZFMK); 1♀, Lishan, Taichung Co., 2020 m, 23/30.IX.1992, coll. F. Aulombard and J. Plante (loaned from ZFMK); 1♂, Hueisun Forest, Nantou Co., 570/800 m, 28/29.IX.1992, coll. F. Aulombard and J. Plante (ZFMK); 1♀, Tien-Hsiang (Hualien Co.), 600 m, 20.VI.1993, coll. F. Aulombard and J. Plante (ZFMK). **Taiwan** (TFRI): 1♀, Taipei Co., Xindian, Wulai, Fushan, 310 m, 9.VI.2013, leg. S. Wu; 2♂, Mioali Co., Nanchuang, Henawan, 850 m, 10.XI.2018, leg. S. Wu; 1♂, Taichung Co., Wuling, 1800 m, 12.IX.2012, leg. S. Wu; 1♂, ditto, 24.X.2014, leg. S. Wu; 1♂, Hualien, Ci’en, 1950 m, 10.XI.2012, leg. S. Wu; 1♀, Chiayi Co., Dabon, 1400 m, 16.VI.2013, leg. S. Wu and W. C. Chang; 1♀, Chiayi Co., Shanmei, 800 m, 9.III.2011, leg. S. Wu and W. C. Chang; 1♀, ditto, 6.X.2011, leg. S. Wu and W. C. Chang; 1♀, Kaohsiung Co., Shanping, 650 m, 3.V.2014, leg. W. C. Liao; 2♂1♀, Taitung Co., Taimali, 26.II.2014, leg. Y. C. Lin.

#### Distribution.

China (Taiwan).

#### Etymology.

The species is dedicated to Dr Dieter Stüning, Bonn, Germany, who has contributed greatly to the taxonomy of the Geometridae.

**Figures 69–74.**
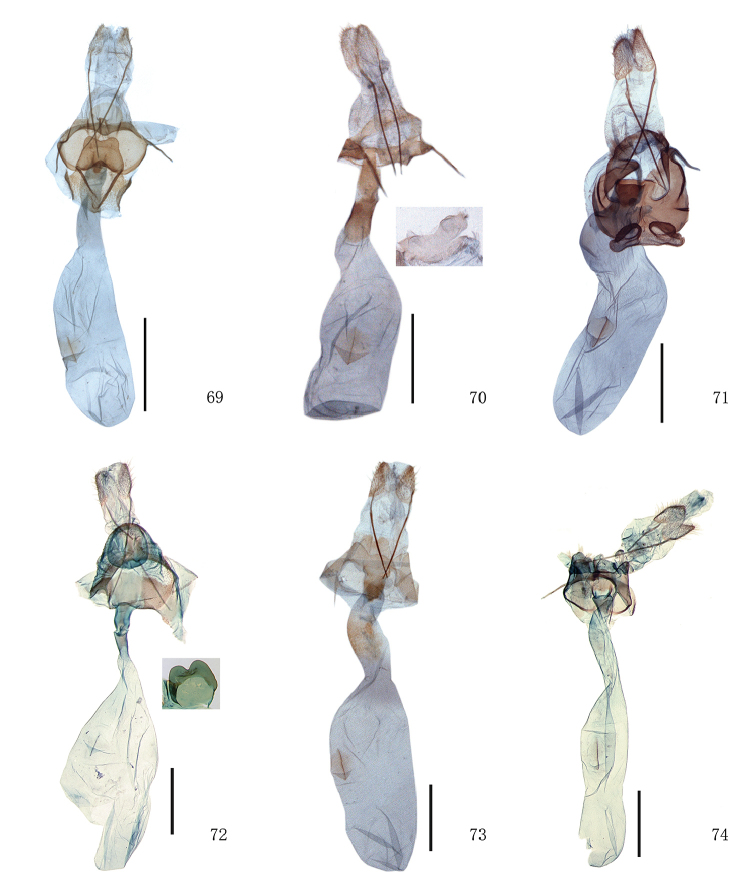
Female genitalia of *Timandra*. **69***T.comptaria*, Yunnan **70***T.viminea* sp. n., Yunnan **71***T.oligoscia*, Hunan **72***T.ruptilinea*, Guangxi **73***T.extremaria*, Zhejiang **74***T.stueningi* sp. n., Taiwan. Scale bar: 1 mm.

## Supplementary Material

XML Treatment for Timandra

XML Treatment for Timandra griseata

XML Treatment for Timandra recompta

XML Treatment for Timandra recompta recompta

XML Treatment for Timandra apicirosea

XML Treatment for Timandra distorta

XML Treatment for Timandra dichela

XML Treatment for Timandra synthaca

XML Treatment for Timandra convectaria

XML Treatment for Timandra correspondens

XML Treatment for Timandra adunca

XML Treatment for Timandra quadrata

XML Treatment for Timandra accumulata

XML Treatment for Timandra comptaria

XML Treatment for Timandra paralias

XML Treatment for Timandra viminea

XML Treatment for Timandra oligoscia

XML Treatment for Timandra ruptilinea

XML Treatment for Timandra extremaria

XML Treatment for Timandra robusta

XML Treatment for Timandra stueningi
